# Doxycycline safety during pregnancy: a large population-based cohort of pregnancies

**DOI:** 10.1007/s15010-025-02622-9

**Published:** 2025-08-22

**Authors:** Itamar Ben Shitrit, Daphna Idan, Ariel Avraham Hasidim, Tal Michael, Amalia Levy, Gali Pariente, Eitan Lunenfeld, Sharon Daniel

**Affiliations:** 1 Joyce and Irving Goldman Medical School, Faculty of Health Sciences, Ben-Gurion University of the Negev, Beer-Sheva, 8410501 Israel; 2 Department of Pediatrics A, Schneider Children’s Medical Center of Israel, Petah Tikva, 94903 Israel; 3 Sackler Faculty of Medicine, Tel Aviv University, Tel Aviv, 6997801 Israel; 4 Department of Epidemiology, Biostatistics, and Community Health Sciences, School of Public Health, Faculty of Health Sciences, Ben- Gurion University of the Negev, Beer-Sheva, Israel; 5 Department of Pediatrics, Faculty of Health Sciences, Ben-Gurion University of the Negev, Beer-Sheva, Israel; 6 Department of Obstetrics and Gynecology, Faculty of Health Sciences, Ben-Gurion University of the Negev and Soroka University Medical Center, Beer-Sheva, Israel; 7 Adelson School of Medicine, Ariel University, Ariel, Israel; 8 Clalit Health Services, Southern District, Beer-Sheva, Israel

**Keywords:** Congenital malformations, Doxycycline, Fetal safety, First trimester infection, Third trimester infection, Prenatal drug exposure

## Abstract

**Purpose:**

Doxycycline is frequently prescribed during pregnancy, yet evidence on fetal safety is inconsistent and often excludes non-live births. We assessed whether exposure during the first or third trimester is associated with major congenital malformations or late-pregnancy adverse outcomes in a population-based cohort that also included stillbirths and terminations.

**Methods:**

Using data from Clalit Health Services Southern district, we identified 265,686 pregnancies in women aged 15–45 years (from 1998 to 2017). Pharmacy records classified doxycycline dispensation in the first trimester (≤ 13 weeks) or third trimester (≥ 27 weeks). Crude and adjusted negative-binomial models estimated relative risks (RRs) for total and organ-specific major congenital malformations diagnosed up to age 1 year and for perinatal mortality, preterm birth, low/very-low birthweight, and low Apgar scores. Sensitivity analyses explored dose-response relations and propensity-score-matched cohorts.

**Results:**

Among 2,696 first-trimester exposures, major malformations occurred in 7.7% versus 7.0% of 262,990 unexposed pregnancies (SMD = 0.03, *p* = 0.17). No association with major malformations was observed in both crude (Crude Relative Risk (RR) = 1.10; 95% CI 0.96–1.27) and adjusted (Adjusted RR = 1.07; 95% CI 0.93–1.23) analyses, nor by organ-specific sub-groups. Third-trimester exposure (*n* = 112) was linked to a higher risk of very-low birthweight, while other late-pregnancy outcomes were comparable to unexposed pregnancies.

**Conclusion:**

First-trimester doxycycline use was not associated with increased major congenital malformation risk, and most late-pregnancy outcomes were unaffected. These findings support the relative safety of doxycycline when clinically indicated during pregnancy.

**Supplementary Information:**

The online version contains supplementary material available at 10.1007/s15010-025-02622-9.

## Introduction

Tetracyclines are broad-spectrum antibiotics [1, 2], widely used for respiratory, skin, and sexually transmitted infections [3], as well as the eradication of Helicobacter pylori [4]. Structural modifications have enhanced their efficacy and safety, resulting in agents such as doxycycline, also indicated for the treatment of zoonotic bacterial diseases such as rickettsia and Brucella [3]. Doxycycline is the predominant agent in its class and ranks as the ninth most commonly prescribed first-trimester medication in a U.S. cohort, with usage rates ranging from 3.8 to 5.3%. In contrast, utilization in Sweden is significantly lower, at only 0.4% [5–9].

Tetracyclines are generally avoided during pregnancy, particularly during the first trimester, due to their ability to cross the placental barrier and affect fetal development [10], and were previously associated with impaired skeletal development, enamel hypoplasia and permanent tooth discoloration [10–12]. Israeli Ministry of Health guidelines also note the risk of tooth discoloration with tetracycline use after the 12th gestational week [13].

Tetracyclines and specifically doxycycline’s safety profile during the first trimester remains ambiguous, with inconsistent literature regarding associations with various congenital anomalies [6, 9, 14–19]. Population-based studies assessing these risks have been constrained by the limited number of exposed pregnancies. For example, Tennessee Medicaid data from 1985 to 2000 identified 1,691 pregnancies exposed to doxycycline [17], while a more recent Swedish cohort specifically reported 4,951 doxycycline-exposed pregnancies between 2006 and 2018 [9]. Also, these studies primarily included live births, excluding pregnancies that ended with induced terminations or stillbirths, a methodological limitation that may underestimate the risk and bias results toward the null hypothesis [20].

The aim of the current study was to examine the associations between doxycycline exposure during the first and third trimesters of pregnancy, and both major congenital malformations and late-pregnancy adverse outcomes, in a large, population- based cohort of pregnancies, including not only live births but also pregnancies that ended in stillbirth or pregnancy terminations, allowing for a more comprehensive assessment of potential risks.

## Methods

We performed a population-based observational retrospective cohort study involving pregnancies of women aged 15 to 45 years who were insured by Clalit Health Services (CHS) health maintenance organization in the southern region of Israel, and delivered or underwent an elective pregnancy termination due to suspected fetal malformation at Soroka University Medical Center (SUMC) between 1998 and 2017. Nearly 70% of childbearing age women in the southern region of Israel are insured by CHS, and about 98% of pregnancies in the region are delivered at SUMC [21]. We excluded pregnancies that were exposed to antimetabolites, isotretinoin, and anti-epileptic drugs, other tetracyclines, as well as those involving multiple gestations or confirmed genetic or chromosomal diagnoses.

### Databases

Data for this study were derived from four distinct databases. Data on deliveries results were obtained from the SMC’s Obstetrics and Gynecology Division’s deliveries database, comprising demographic information, diagnoses during pregnancy and delivery results. Data concerning congenital malformations were retrieved by SMC’s hospitalization database, documenting major congenital malformations diagnosed from birth and up to one year of age. All medical diagnoses were assigned by board-certified neonatologists or pediatricians and were coded according to the International Classification of Diseases, 9th Revision (ICD-9). Information on malformations diagnosed before elective pregnancy terminations, performed due to suspected fetal malformation, was manually extracted from the registry of the Committee for Termination of Pregnancies at SMC. These data encompassed maternal and pregnancy characteristics, as well as major fetal malformations identified through ultrasound or echocardiography. Medication dispensation records were obtained from the CHS database, providing details on dispensed medications before and during pregnancy, including drug names, Anatomical Therapeutic Chemical (ATC) classification codes, and the quantity dispensed in defined daily doses (DDD).

The databases were integrated into a single dataset using the Israeli Identification Number, which is uniquely assigned to each citizen at birth by the Ministry of the Interior, and a unique hospitalization number assigned to each admission at SMC. These identifiers facilitated the linkage of maternal records with corresponding newborn or elective pregnancy termination data.

The research was approved by SMC’s ethics committee in accordance with the Declaration of Helsinki (study number: 0069–20-SOR, Approval date: 07/03/2022).

### Definition of variables

The gestational age at birth was documented and verified for every pregnancy, including live births and pregnancy terminations, by board-certified gynecologists, based on the first day of the last menstrual period or ultrasound-based gestational age assessment.

#### Exposure

We defined two exposure groups: pregnancies in which doxycycline were dispensed from the first day of the last menstrual period until the end of the 13th gestational week were classified as the first-trimester exposure group, while those with exposure from the 27th gestational week until the end of pregnancy were classified as the third-trimester exposure group.

Exposure was also defined in terms of the number of DDD dispensed during the first and third trimester. We categorized doxycycline exposure (DDD = 100 mg) into four groups: unexposed, short-term (1–7 DDD), medium-term (8–28 DDD), and long-term (> 28 DDD).

#### Major congenital malformations

We defined major congenital malformations according to the Metropolitan Atlanta Congenital Defects Program of the Centers for Disease Control and Prevention. Malformations were classified according to the International Classification of Diseases, 9th Revision (ICD-9), into the following categories: anencephaly (ICD-9 code 740), spina bifida (ICD-9 code 741), other nervous system anomalies (ICD-9 code 742), eye anomalies (ICD-9 code 743), anomalies of the ear, face, and neck (ICD-9 code 744), bulbus cordis and cardiac septal closure anomalies (ICD-9 code 745), other heart anomalies (ICD-9 code 746), other circulatory system anomalies (ICD-9 code 747), respiratory system anomalies (ICD-9 code 748), cleft palate and cleft lip (ICD-9 code 749), anomalies of the upper alimentary tract (ICD-9 code 750), other digestive system anomalies (ICD-9 code 751), genital anomalies (ICD-9 code 752), urinary system anomalies (ICD-9 code 753), musculoskeletal deformities (ICD-9 code 754), limb anomalies (ICD-9 code 755), other musculoskeletal anomalies (ICD-9 code 756), and integumentary system anomalies (ICD-9 code 757).

We assessed associations between first trimester exposure to doxycycline and the total number of major congenital malformations, and for the following groups of malformations according to organ systems: cardiovascular (CVS) malformations (codes 745, 746, 747), central nervous system (CNS) malformations (codes 740, 741, 742, 743), musculoskeletal (MSK) malformations (codes 754, 755, 756), gastrointestinal (GI) malformations (codes 750, 751) and genitourinary (GU) malformations (codes 752, 753).

#### Late pregnancy adverse outcomes

Late adverse pregnancy outcomes were defined as follows: low birth weight (< 2500 g), very low birth weight (< 1500 g), low Apgar scores at 1 min < 7, low Apgar scores at 5 min < 7, perinatal death (antepartum death [APD], intrapartum death [IPD] and postpartum death [PPD]) and preterm birth (birth before the 37th gestational week).

### Statistical analyses

Statistical analyses were performed using R (version 4.4.3). Maternal characteristics and late pregnancy outcomes were compared between pregnancies exposed and unexposed to doxycycline during the first and third trimesters using the χ² test for categorical variables, Student’s t-test for normally distributed continuous variables, and the Mann-Whitney test for ordinal or non-normally distributed continuous variables.

To assess the independent risk for major congenital malformations and organ system-specific malformations following first-trimester doxycycline exposure, we utilized both univariate and multivariate negative-binomial regression models. Relative risks (RRs) and 95% confidence intervals (CIs) were calculated. Confounders controlled for in the major malformation models included maternal age, ethnicity (Jewish vs. Bedouin), birth order, child sex, gestational age at birth, and year of birth or pregnancy termination.

Due to the relatively small second and third-trimester exposure groups (*n* = 134 and *n* = 112 exposed to doxycycline during the 2nd and 3rd trimesters, respectively), we did not calculate relative risks for late adverse pregnancy outcomes.

As a sensitivity analysis, we constructed similar models evaluating exposure based on defined daily dose (DDD) groups, categorizing users into unexposed, short-term (1–7 DDD), medium-term (8–28 DDD), and long-term (> 28 DDD) doxycycline exposure cohorts.

As an additional sensitivity analysis, we performed k-nearest neighbor propensity score matching, pairing any doxycycline-exposed with unexposed pregnancies at a 1:10 ratio using a caliper of 0.1. Matching covariates included maternal age, ethnicity (Jewish vs. Bedouin), gestational age at birth, number of births, and year of birth or pregnancy termination. Subsequently, we assessed the risk for major congenital malformations among the matched vs. the unmatched pregnancies using univariate negative binomial regression models.

Standardized Mean Differences (SMDs) were used to compare groups, with values 0.2, 0.5, and 0.8 are considered small, medium, and large differences, respectively [22]. All statistical analyses were conducted at a significance level of α = 0.05 (two-sided).

## Results

### Study population and demographic characteristics

**Fig. 1:**
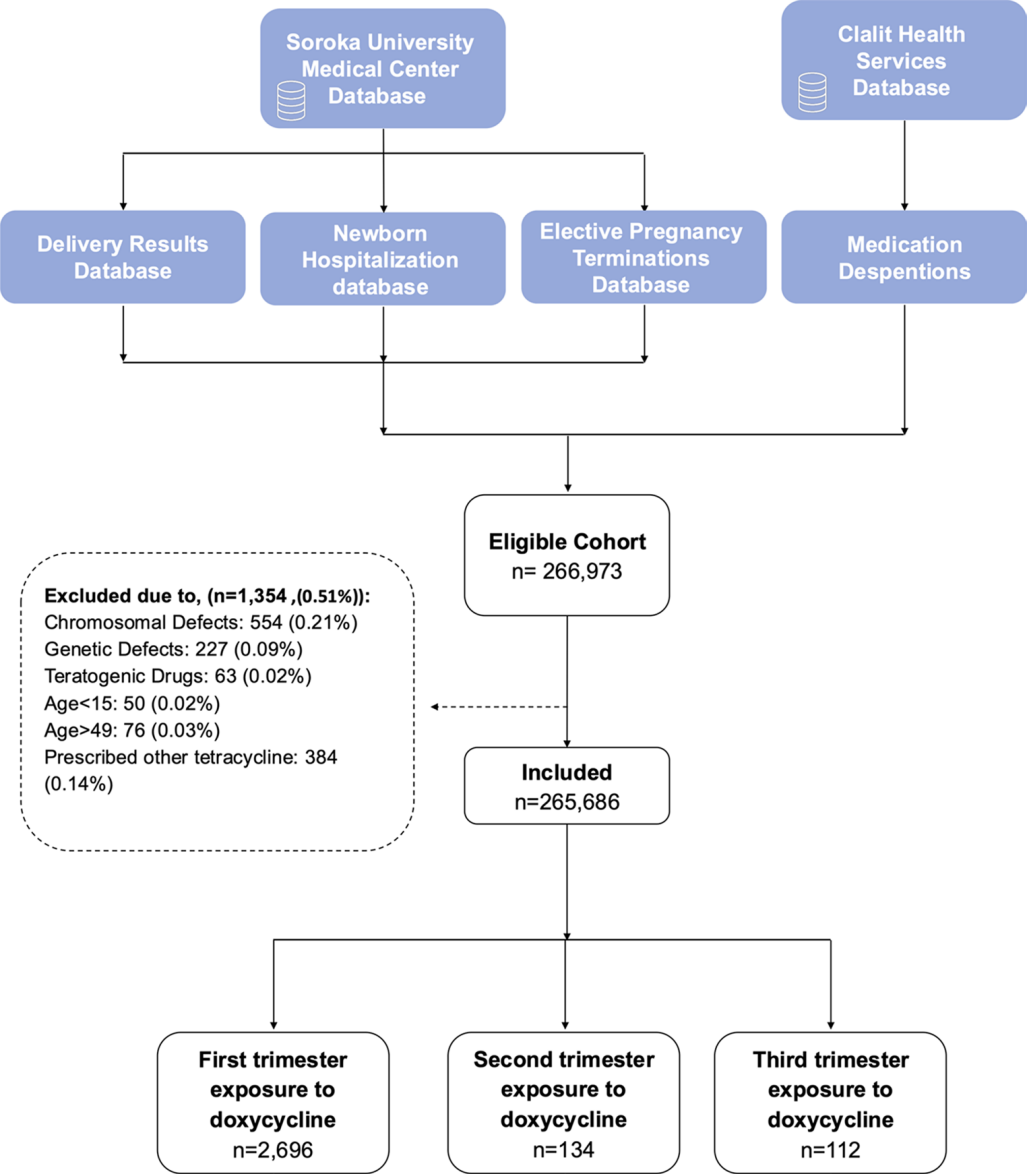
Flow chart of study groups. This figure presents the number of women included in the study cohort after the exclusion criteria among the exposed and unexposed groups according to the different trimesters of pregnancy pre and post matching

Between 1998 and 2017, a total of 266,973 pregnancies among women insured by Clalit Health Services resulted in birth or pregnancy termination at SUMC. Of these, 265,686 pregnancies met the inclusion criteria. A total of 2,696 (1.01%), 134 (0.05%) and 112 (0.04%) were exposed to doxycycline during the first, second and third trimesters, respectively (Fig. 1).

Table 1 presents a comparison of maternal characteristics between first trimester exposed and unexposed pregnancies. A higher proportion of Bedouin mothers were exposed to doxycycline compared to Jewish mothers (80% vs. 55%, SMD = 0.56, *p* < 0.001). Median gestational age at delivery was similar (276 days [IQR 266–282] vs. 275 days [266–281], SMD = 0.08, *p* = 0.041). Doxycycline-exposed mothers were more likely to have had five or more births (38% vs. 24%, SMD = 0.32, *p* < 0.001).

**Table 1:** Comparison of maternal characteristics among pregnancies exposed vs. unexposed to doxycycline during the first trimester

Maternal characteristics	First trimester exposure to doxycycline		Difference^2^	*p*-value^3^
	Yes = 2,696^1^	No = 262,990^1^		
Mother’s age, years	28.6 (24.7, 33.0)	28.4 (24.4, 32.9)	0.02	0.16
Ethnic group (Bedouin)	2,154 (80%)	144,208 (55%)	0.55	<0.001
Gestational age at birth, days	276 (266, 282)	275 (266, 281)	0.08	0.041
Gestational age at birth, weeks	39.40 (38.00, 40.30)	39.30 (38.00, 40.10)	0.08	0.041
Number of Births			0.32	<0.001
1	453 (17%)	64,743 (25%)		
2-4	1,216 (45%)	134,252 (51%)		
≥5	1,025 (38%)	63,247 (24%)		
Smoking during pregnancy	13 (0.5%)	1,030 (0.4%)	0.01	0.45
Pre-gestational diabetes	30 (1.1%)	972 (0.4%)	0.09	<0.001
Gestational diabetes	9 (0.3%)	310 (0.1%)	0.05	0.006
Sex of newborn (males)	1,391 (51%)	134,163 (51%)	0.00	0.93
Year of delivery/elective pregnancy termination	2,007 (2,003, 2,012)	2,008 (2,003, 2,013)	0.15	<0.001
Pregnancy elective termination	1 (<0.1%)	1825 (0.7%)	0.11	<0.001

^1^Median (Q1, Q3); n (%)

^2^absolute Standardized Mean Difference

^3^Wilcoxon rank sum test; Pearson’s Chi-squared test

### Major malformations

**Fig. 2:**
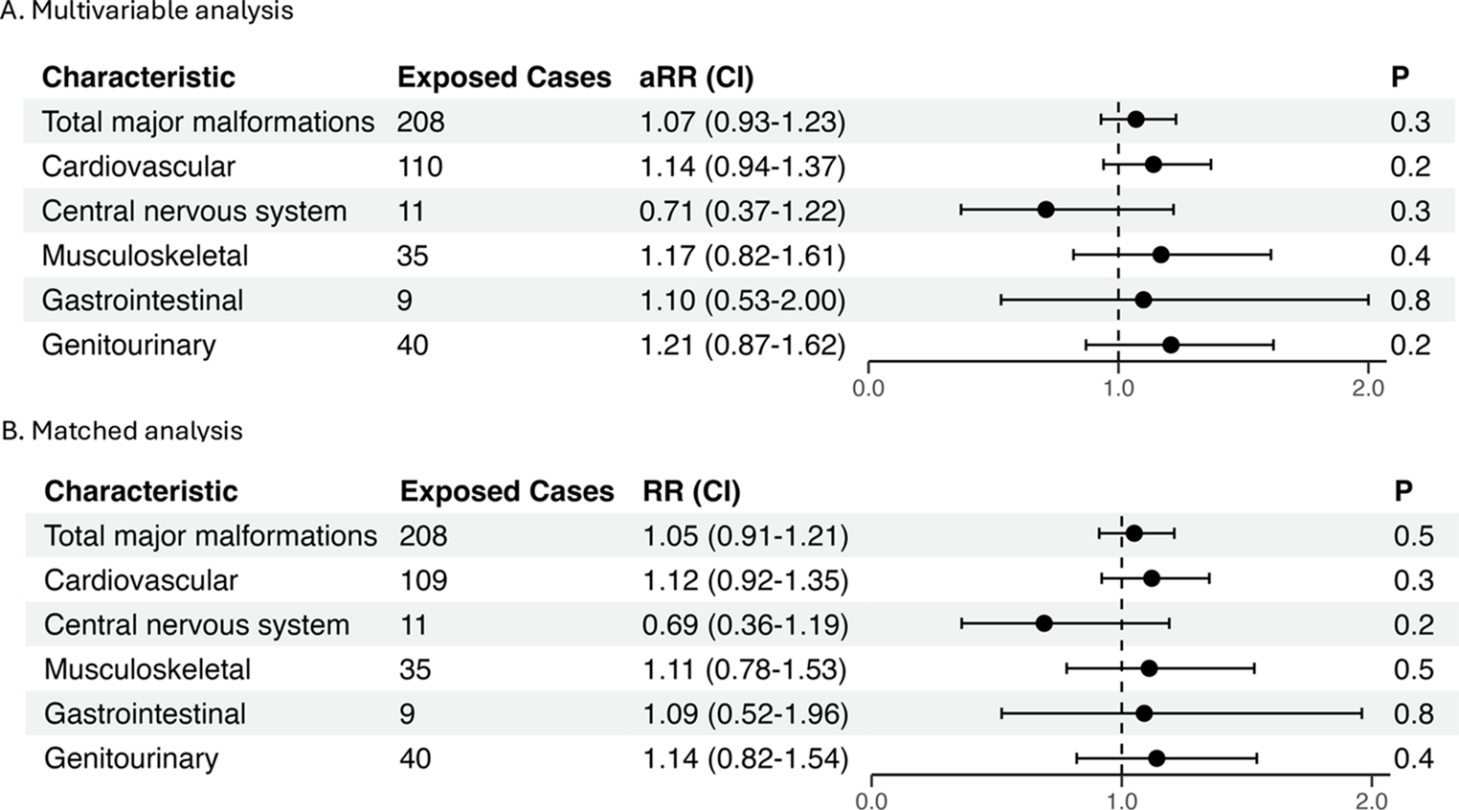
Risk of total major malformations and malformations by organ systems in (**A**) Adjusted* negative regression models and (**B**) propensity score matching*. Models were adjusted for maternal age, ethnicity (Jewish vs. Bedouin), birth order, child sex, gestational age at birth, and year of birth or pregnancy termination. CVS: Cardiovascular system, CNS: Central Nervous system, MS: Musculoskeletal, GI: Gastrointestinal, GU: Genitourinary

Among 2,696 doxycycline-exposed pregnancies during the first trimester, 208 (7.7%) were diagnosed with major malformations, compared with 18,447 (7.0%) of unexposed pregnancies (SMD = 0.03, *p* = 0.17, Table 2). No association with major malformations was observed in both crude (Crude Relative Risk (RR) = 1.10; 95% CI 0.96–1.27, Table 2), and adjusted (Adjusted RR = 1.07; 95% CI 0.93–1.23, exposed cases = 208, Fig. 2, Table S1) analyses. Specifically, cardiovascular malformations were more prevalent among exposed pregnancies (4.1% vs. 3.3%, SMD = 0.04, *p* = 0.023, aRR = 1.25; 95% CI 1.02–1.50, Table 2; Fig. 2, Table S1), however, no significant associations were found for malformations according to organ systems (cardiovascular, central nervous, musculoskeletal, gastrointestinal, genitourinary systems and cleft palate) after adjustment (Table 2; Fig. 2).

**Table 2:** Total major malformations and malformations by organ systems following first trimester exposure to doxycycline- frequencies and crude negative binomial regression results

Malformations by organ systems	Frequencies				Univariable regression	
	First trimester exposure to doxycycline *N* = 2,696^1^	Unexposed *N* = 262,990^1^	Difference (SMD)^2^	*p*-value^3^	RR^4^	95% CI^5^
Total major malformations	208 (7.7%)	18,447 (7.0%)	0.03	0.17	1.1	0.96, 1.27
Cardiovascular	110 (4.1%)	8,655 (3.3%)	0.04	0.023	1.25	1.02, 1.50
Central nervous system	11 (0.4%)	1,530 (0.6%)	0.02	0.23	0.7	0.36, 1.20
Musculoskeletal	35 (1.3%)	2,851 (1.1%)	0.02	0.28	1.2	0.84, 1.64
Gastrointestinal	9 (0.3%)	760 (0.3%)	0.01	0.66	1.15	0.55, 2.08
Genitourinary	40 (1.5%)	3,360 (1.3%)	0.02	0.34	1.16	0.84, 1.56
Cleft Palate	3 (0.1%)	294 (0.1%)	0	1	1.00	--,--

^1^n (%)

^2^absolute Standardized Mean Difference

^3^Pearson’s Chi-squared test; Fisher’s exact test

^4^Negative binomial regression

^5^CI; Confidence Interval

## Sensitivity analyses

### Analyses by DDD

A total of 285, 2,255 and 144 pregnancies were exposed to short-term (1–7), medium-term (8–28) and log-term (> 28) doxycycline treatment. Compared with unexposed pregnancies, there was no significant dose response between exposure to doxycycline in terms of the DDD dispensed, and major malformations (Table 3, Table S2).

**Table 3:** Risk for total major congenital malformations following first-trimester exposure to Doxycycline as defined daily doses (DDD): results of adjusted negative binomial regression models

Variable	Exposed cases of major malformations^1^	aRR^1^	95% CI^1^	*p*-value
First Trimester DDD Doxycycline Exposure				
Unexposed	18,447/262,990, (7.0%)	—	—	—
1-7 DDD	17/2,696, (0.6%)	1.16	0.46, 2.36	0.7
8-28 DDD	180/2,696, (6.6)%	0.82	0.52, 1.22	0.4
>28 DDD	10/2,696, (0.37%)	1.58	0.49, 3.68	0.4

^1^n (%), aRR = adjusted Risk Ratio, CI = Confidence Interval

### Propensity score matching

In propensity score matching, a cohort comprising 2,687 exposed and 26,870 unexposed pregnancies (99.5% of pregnancies exposed to doxycycline from the full cohort) was assembled (Figure S1). Maternal characteristics were similar between the groups, except for the number of past pregnancies and pre-gestational diabetes mellitus (Table S3). Overall, exposure to doxycycline was not associated with an increased risk of major malformations (7.7% exposed cases vs. 7.4% non-exposed cases; SMD = 0.01; *p* = 0.47; RR = 1.05; 95% CI 0.91–1.21; *p* = 0.5, Table S4).

### Late pregnancy adverse outcomes following third trimester exposure

Late pregnancy adverse outcomes were not significantly different between pregnancies exposed and unexposed to doxycycline during the third trimester, with similar rates of low birth weight (SMD = 0.04, *p* = 0.65), Apgar scores < 7 at 1 min (SMD = 0.08, *p* = 0.63) and 5 min (SMD = 0.02, *p* = 0.54), perinatal death (SMD = 0.11, *p* = 0.13), and preterm delivery (SMD = 0.05, *p* = 0.61). However, very low birth weight was more common among exposed pregnancies (*n* = 6, 5.4% vs. *n* = 3,397, 1.3%, SMD = 0.23, *p* = 0.003) (Table S5).

Second-trimester doxycycline exposure was also not significantly associated with adverse pregnancy outcomes (Table S6).

## Discussion

### Principal findings

In this large population-based cohort study of nearly 2,700 singleton infants exposed to doxycycline, first-trimester doxycycline exposure was not associated with increased risks of any major malformation overall or by organ system, also no dose-response effect was apparent even among pregnancies exposed during a period of more than one month compared with unexposed pregnancies. An increased unadjusted risk for cardiovascular malformations was observed; however, this association was insignificant following adjustment for potential confounders. These findings were consistent in analyses using propensity score matching, further strengthening the robustness of the results.

Smoking was not associated with doxycycline exposure and was therefore not included as a covariate in the primary analysis. Gestational diabetes was also not included due to its correlation with pregestational diabetes. Nonetheless, we performed sensitivity analyses adjusting for smoking and gestational diabetes, which yielded similar results.

We found no differences in the rates of late adverse pregnancy outcomes between pregnancies exposed and unexposed during the third trimester, except for a higher rate of very low birthweight among exposed pregnancies. However, this observation should be interpreted with caution, as it is based on a relatively small number of third-trimester exposures.

Doxycycline treats a wide spectrum of infections, including sexually transmitted infections like chlamydia and syphilis [3]—conditions independently associated with adverse pregnancy outcomes [23–26]. In such cases, any observed association between doxycycline exposure and congenital malformations could potentially reflect confounding by indication, artificially inflating risk estimates. Notably, despite this potential bias, our findings demonstrated no association, which may provide reassurance regarding doxycycline use during pregnancy.

### Results in the context of what is known

As Anker notes in his commentary on Nakitanda et al.‘s study, “pregnant persons still need to be viewed as therapeutic orphans,” highlighting the persistent lack of robust safety data on tetracycline use during pregnancy [18]. Our research advances the existing literature by examining more precise dosage groups (1–7 days, 8–28 days, and > 28 days) [9], enabling a more nuanced evaluation of potential dose-related effects. Additionally, we included data from electively terminated pregnancies—a population often excluded from observational studies of teratogenic risk [6, 9, 15, 17]—yet found no evident signal of increased risk in this subgroup. The prevalence of major congenital malformations in our study was higher than previously reported (approximately 7% vs. the 3–4% reported in population-based studies) [27, 28]. This discrepancy may be attributed to several methodological and population-specific factors. First, our analysis included malformations diagnosed up to one year after delivery, capturing anomalies that may not be apparent at birth. Second, we included cases identified in elective terminations of pregnancy following prenatal diagnosis, which are often underrepresented in studies restricted to live births [20]. Lastly, our cohort comprised a significant proportion of women of Bedouin origin, a population with a high rate of consanguineous marriages [29], likely contributing to the increased congenital anomalies rate. Our findings align with several previously published research. We observed no significant association between doxycycline exposure during the first trimester, and overall major malformations (exposed 2,696; aRR = 1.07; 95% CI 0.93–1.23), which closely corresponds with the results from the Swedish cohort (exposed 4,956; matched RR = 1.07; 95% CI 0.93–1.23) [9]. Our results are also congruent with studies from Canada (exposed 164; OR, 1.04; 95% CI 0.75–1.43) [15], the US (1,691 exposed; RR 0.85; 95% CI 0.59–1.23) [17], and Denmark (1,101 exposed, aOR; 1.10; 95% CI 0.85–1.41) [6, 9, 16]. Notably, the Canadian study [15] reported an increased risk of cardiac malformations, however, those results were based on only 9 exposed cases, in contrast to our analysis, which included 110 exposed cases. Also, our data contained malformations diagnosed up to one year of age and malformations diagnosed before induced pregnancy terminations due to suspected fetal- malformations.

### Clinical implications

Our findings provide reassuring evidence regarding the safety of doxycycline use in early pregnancy, showing no increased risk of major congenital malformations—even with extended exposure. Given doxycycline’s broad-spectrum efficacy, including against sexually transmitted infections that independently pose risks to pregnancy, these results support more confident clinical decision-making, especially in situations where alternative antibiotics may be less suitable or unavailable.

### Research implications

Incorporating data from elective pregnancy terminations, as done in our study, is crucial for capturing a more comprehensive range of outcomes and reducing selection bias. Larger studies with greater sample sizes are needed to validate our findings, particularly for rare malformation subtypes and late pregnancy outcomes.

### Strengths and limitations

Several methodological limitations must be acknowledged. Our exposure classification was based on dispensation records rather than verified medication consumption, which may introduce exposure misclassification. However, prescription databases have demonstrated a high correlation with actual medication use in the general population and specifically among pregnant women [30–32] and have been validated as reliable data sources for studying associations between pharmacological agents and congenital malformations [33]. Additionally, our dataset lacked information regarding specific prescription indications, limiting our ability to account for potential confounding by indication or to identify appropriate active comparators. However this limitation was reported in all previously reported data [6, 9, 15, 17]. Nevertheless, confounding by indication would unlikely bias results toward the null, as underlying conditions for which the study medication is prescribed would more plausibly increase rather than decrease malformation risk [26]. Another limitation of our study is the relatively small number of pregnancies exposed to doxycycline during the third trimester. As a result, findings related to late pregnancy exposure, including the observed increase in very low birthweight, should be interpreted with caution due to limited statistical power. Also, since data on tooth staining, a known potential adverse effect of tetracycline use in late pregnancy, were not available, we were unable to assess the risk for this specific outcome in our study.

This study represents one of the largest cohorts to date examining the association between doxycycline exposure and congenital malformations, and includes the second-largest dataset on this topic. Drawing on data covering approximately 70% of all pregnancies in the southern district [21], the study offers strong population-level representation. The use of pharmacy dispensation records, rather than prescription data, enhances the accuracy of exposure assessment by better capturing actual medication use. Additionally, by including outcomes from live births, stillbirths, and elective pregnancy terminations, the study mitigates potential bias toward the null - a limitation noted in previous research [20]. Malformations were diagnosed by trained neonatologists and tracked up to one year of age, allowing for more complete ascertainment of outcomes.

## Conclusion

Exposure to doxycycline during the first trimester was not associated with overall major congenital malformations or specific organ system malformations in the population studied, compared with non-exposed pregnancies. These findings support prior evidence suggesting the relative safety of doxycycline in early pregnancy. Third-trimester findings, including a higher rate of very low birthweight, should be interpreted with caution due to the small number of exposed cases.

We believe these findings have significant clinical relevance given the importance of effective antibiotic options during pregnancy.

## Supplementary Information

Below is the link to the electronic supplementary material.

Supplementary Material 1

## Data Availability

Data is available on reasonable request from the corresponding author, subject to approval of the Institutional Review Board Committee of the Soroka University Medical Center.

## Declarations

### Tweetable statement

Large cohort supports evidence that first-trimester doxycycline exposure does not increase major congenital malformations, even when stillbirths and terminations are included.
