# Correction: Doxycycline safety during pregnancy: a large population-based cohort of pregnancies

**DOI:** 10.1007/s15010-025-02641-6

**Published:** 2025-10-09

**Authors:** Itamar Ben Shitrit, Daphna Idan, Ariel Avraham Hasidim, Tal Michael, Amalia Levy, Gali Pariente, Eitan Lunenfeld, Sharon Daniel

**Affiliations:** 1 Joyce and Irving Goldman Medical School, Faculty of Health Sciences, Ben-Gurion University of the Negev, Beer-Sheva, 8410501 Israel; 2 Department of Pediatrics A, Schneider Children’s Medical Center of Israel, Petah Tikva, 94903 Israel; 3 Sackler Faculty of Medicine, Tel Aviv University, Tel Aviv, 6997801 Israel; 4 Department of Epidemiology, Biostatistics, and Community Health Sciences, School of Public Health, Faculty of Health Sciences, Ben-Gurion University of the Negev, Beer-Sheva, Israel; 5 Department of Pediatrics, Faculty of Health Sciences, Ben-Gurion University of the Negev, Beer-Sheva, Israel; 6 Department of Obstetrics and Gynecology, Faculty of Health Sciences, Ben-Gurion University of the Negev and Soroka University Medical Center, Beer-Sheva, Israel; 7 Adelson School of Medicine, Ariel University, Ariel, Israel; 8 Clalit Health Services, Southern District, Beer-Sheva, Israel

**Infection**

10.1007/s15010-025-02622-9

In this article the author’s name Ariel Avraham Hasidim was incorrectly written as Ariel Avraham Hassidim.

The Original Article has been corrected.

