# Supplementary material for: Doxycycline safety during pregnancy: a large population-based cohort of pregnancies
Source: Infection. 2025 Aug 22;53(6):2739–47. doi: 10.1007/s15010-025-02622-9 (PMC12675617; doi:10.1007/s15010-025-02622-9)
Supplement: Supplementary file 1 — Supplementary Material 1 [file 15010_2025_2622_MOESM1_ESM.docx]

**Supplementary Chapter 1 - Main analysis**

Table S1. Multivariable Negative Binomial Regression for Major Malformations

|  | **Major malformations total** | | | **Cardiovascular** | | | **Neurological** | | | **Musculoskeletal** | | | **Gastrointestinal** | | | **Urogenital** | | | **Cleft Palate** | | |
| --- | --- | --- | --- | --- | --- | --- | --- | --- | --- | --- | --- | --- | --- | --- | --- | --- | --- | --- | --- | --- | --- |
| **Variable** | **aRR**^1^ | **95% CI**^1^ | **p-value** | **aRR**^1^ | **95% CI**^1^ | **p-value** | **aRR**^1^ | **95% CI**^1^ | **p-value** | **aRR**^1^ | **95% CI**^1^ | **p-value** | **aRR**^1^ | **95% CI**^1^ | **p-value** | **aRR**^1^ | **95% CI**^1^ | **p-value** | **aRR**^1^ | **95% CI**^1^ | **p-value** |
| First Trimester Doxycycline Exposure | 1.07 | 0.93, 1.23 | 0.3 | 1.14 | 0.94, 1.37 | 0.2 | 0.71 | 0.37, 1.22 | 0.3 | 1.17 | 0.82, 1.61 | 0.4 | 1.10 | 0.53, 2.00 | 0.8 | 1.21 | 0.87, 1.62 | 0.2 | 0.83 | 0.21, 2.18 | 0.8 |
| Mother's age, years | 1.01 | 1.01, 1.01 | <0.001 | 1.01 | 1.01, 1.02 | <0.001 | 1.00 | 0.99, 1.01 | 0.7 | 1.01 | 1.00, 1.02 | 0.011 | 1.02 | 1.00, 1.03 | 0.050 | 1.00 | 1.00, 1.01 | 0.4 | 0.99 | 0.97, 1.02 | 0.7 |
| Gestational age, weeks | 0.89 | 0.89, 0.89 | <0.001 | 0.92 | 0.91, 0.92 | <0.001 | 0.79 | 0.78, 0.80 | <0.001 | 0.96 | 0.95, 0.98 | <0.001 | 0.86 | 0.84, 0.88 | <0.001 | 0.91 | 0.90, 0.92 | <0.001 | 0.94 | 0.90, 0.99 | 0.008 |
| Sex of newborn (males) | 1.28 | 1.25, 1.32 | <0.001 | 1.02 | 0.98, 1.07 | 0.3 | 0.92 | 0.83, 1.03 | 0.14 | 1.02 | 0.95, 1.10 | 0.5 | 0.66 | 0.57, 0.76 | <0.001 | 7.49 | 6.74, 8.36 | <0.001 | 1.12 | 0.89, 1.41 | 0.3 |
| Birth order |  |  |  |  |  |  |  |  |  |  |  |  |  |  |  |  |  |  |  |  |  |
| ≥5 | — | — |  | — | — |  | — | — |  | — | — |  | — | — |  | — | — |  | — | — |  |
| 1 | 1.07 | 1.01, 1.13 | 0.016 | 0.89 | 0.82, 0.96 | 0.003 | 0.95 | 0.78, 1.15 | 0.6 | 1.64 | 1.43, 1.88 | <0.001 | 1.04 | 0.80, 1.34 | 0.8 | 1.19 | 1.05, 1.35 | 0.007 | 0.71 | 0.46, 1.09 | 0.12 |
| 2-4 | 0.96 | 0.92, 1.00 | 0.074 | 0.90 | 0.85, 0.96 | <0.001 | 0.92 | 0.79, 1.07 | 0.3 | 1.13 | 1.01, 1.27 | 0.027 | 0.88 | 0.72, 1.08 | 0.2 | 1.07 | 0.97, 1.18 | 0.2 | 0.85 | 0.62, 1.17 | 0.3 |
| Ethnic group (Bedouin) | 1.23 | 1.19, 1.28 | <0.001 | 1.36 | 1.30, 1.44 | <0.001 | 2.17 | 1.89, 2.49 | <0.001 | 1.50 | 1.37, 1.64 | <0.001 | 1.30 | 1.10, 1.53 | 0.003 | 1.00 | 0.92, 1.08 | >0.9 | 2.19 | 1.63, 2.96 | <0.001 |
| Year of delivery/elective pregnancy termination | 1.00 | 1.00, 1.01 | 0.005 | 1.01 | 1.01, 1.02 | <0.001 | 1.02 | 1.01, 1.03 | <0.001 | 1.00 | 1.00, 1.01 | 0.2 | 1.04 | 1.02, 1.05 | <0.001 | 1.02 | 1.01, 1.02 | <0.001 | 1.01 | 0.99, 1.03 | 0.6 |
| ^1^aRR = adjusted Risk Ratio, CI = Confidence Interval | | | | | | | | | | | | | | | | | | | | | |

Supplementary Chapter 2 - Sensitivity Analysis

Defined daily dose (DDD) analysis

Table S2. The risk (adjusted relative risk [aRR] and 95% confidence interval [95% CI]) of total major congenital malformations following first-trimester exposure to Doxycycline as defined daily dose (DDD): results of multivariate negative binomial regression model

| **Variable** | **aRR**^1^ | **95% CI**^1^ | **p-value** |
| --- | --- | --- | --- |
| First Trimester DDD Doxycycline Exposure |  |  |  |
| 0 DDD | — | — |  |
| 0-7 DDD | 0.80 | 0.48, 1.24 | 0.4 |
| 8-28 DDD | 1.11 | 0.95, 1.28 | 0.2 |
| >28 DDD | 1.06 | 0.53, 1.86 | 0.9 |
| Mother's age, years | 1.01 | 1.01, 1.01 | <0.001 |
| Gestational age, weeks | 0.89 | 0.89, 0.89 | <0.001 |
| Sex of newborn (males) |  |  |  |
| Female | — | — |  |
| Male | 1.28 | 1.25, 1.32 | <0.001 |
| Birth order |  |  |  |
| ≥5 | — | — |  |
| 1 | 1.07 | 1.01, 1.13 | 0.016 |
| 2-4 | 0.96 | 0.92, 1.00 | 0.075 |
| Ethnic group (Bedouin) | 1.23 | 1.19, 1.28 | <0.001 |
| Year of delivery/elective pregnancy termination | 1.00 | 1.00, 1.01 | 0.005 |
| ^1^aRR = adjusted Risk Ratio, CI = Confidence Interval | | | |


Table S3. Matched cohort Comparison of maternal characteristics among exposed vs. unexposed to any doxycycline during the first trimester

| **Matched cohort - Maternal characteristics** | **First trimester exposure to doxycycline**  N = 2,687^1^ | **Unexposed**  N = 26,870^1^ |  | **Difference**^2^ |  | **p-value**^3^ |
| --- | --- | --- | --- | --- | --- | --- |
| Mother's age, years | 28.6 (24.7, 33.0) | 28.5 (24.6, 32.9) |  | -0.01 |  | 0.47 |
| Ethnic group (Bedouin) | 2,148 (80%) | 21,477 (80%) |  | 0.00 |  | 0.98 |
| Number of Births |  |  |  | 0.08 |  | <0.001 |
| 1 | 449 (17%) | 3,859 (14%) |  |  |  |  |
| 2-4 | 1,214 (45%) | 13,153 (49%) |  |  |  |  |
| ≥5 | 1,024 (38%) | 9,858 (37%) |  |  |  |  |
| Gestational age , days | 276 (266, 282) | 277 (266, 282) |  | 0.01 |  | 0.40 |
| Gestational age, weeks | 39.40 (38.00, 40.30) | 39.60 (38.00, 40.30) |  | 0.01 |  | 0.40 |
| Smoking during pregnancy | 13 (3.1%) | 75 (2.4%) |  | -0.04 |  | 0.42 |
| Pre-gestational diabetes | 30 (7.1%) | 114 (3.7%) |  | -0.15 |  | <0.001 |
| Sex of newborn (males) | 1,378 (51%) | 13,867 (52%) |  | 0.01 |  | 0.74 |
| Year of delivery/elective pregnancy termination | 2,007 (2,003, 2,012) | 2,007 (2,003, 2,012) |  | 0.01 |  | 0.80 |
| Pregnancy Termination | 0 (0%) | 0 (0%) |  | 0.00 |  | -- |
| ^1^Median (Q1, Q3); n (%)  ^2^Standardized Mean Difference  ^3^Wilcoxon rank sum test; Pearson's Chi-squared test | | | | | | |

Table S4. Matched cohort, comparison of congenital malformations among exposed vs. unexposed to any doxycycline during the first trimester

| **Malformations by organ systems** | **Frequencies** | | | | | **Univariable regression (exposure to doxycyline during first trimester)** | | |
| --- | --- | --- | --- | --- | --- | --- | --- | --- |
|  | **First trimester exposure to doxycycline**  N = 2,687^1^ | **Unexposed**  N = 26,870^1^ | **Difference**^2^ | **p-value**^3^ |  | **RR**^4^ | **95% CI**^5^ | **p-value6**^6^ |
| Total major malformations | 208 (7.7%) | 1,979 (7.4%) | 0.01 | 0.47 |  | 1.05 | 0.91, 1.21 | 0.5 |
| Cardiovascular | 109 (4.1%) | 968 (3.6%) | 0.01 | 0.23 |  | 1.12 | 0.92, 1.35 | 0.3 |
| Central nervous system | 11 (0.4%) | 163 (0.6%) | 0.03 | 0.20 |  | 0.69 | 0.36, 1.19 | 0.2 |
| Musculoskeletal | 35 (1.3%) | 311 (1.2%) | 0.01 | 0.50 |  | 1.11 | 0.78, 1.53 | 0.5 |
| Gastrointestinal | 9 (0.3%) | 82 (0.3%) | 0.01 | 0.70 |  | 1.09 | 0.52, 1.96 | 0.8 |
| Genitourinary | 40 (1.5%) | 82 (0.3%) | 0.02 | 0.38 |  | 1.14 | 0.82, 1.54 | 0.4 |
| Cleft Palate | 3 (0.1%) | 40 (0.1%) | 0.01 | 0.79 |  | 1.00 | --,-- | -- |
| ^1^n (%)  ^2^Standardized Mean Difference  ^3^Pearson's Chi-squared test; Fisher's exact test  ^4^Negative binomial regression, RR; Risk Ratio  ^5^CI; Confidence Interval  ^6^Qassi-Poisson regression | | | | | | | | |

Figure S1


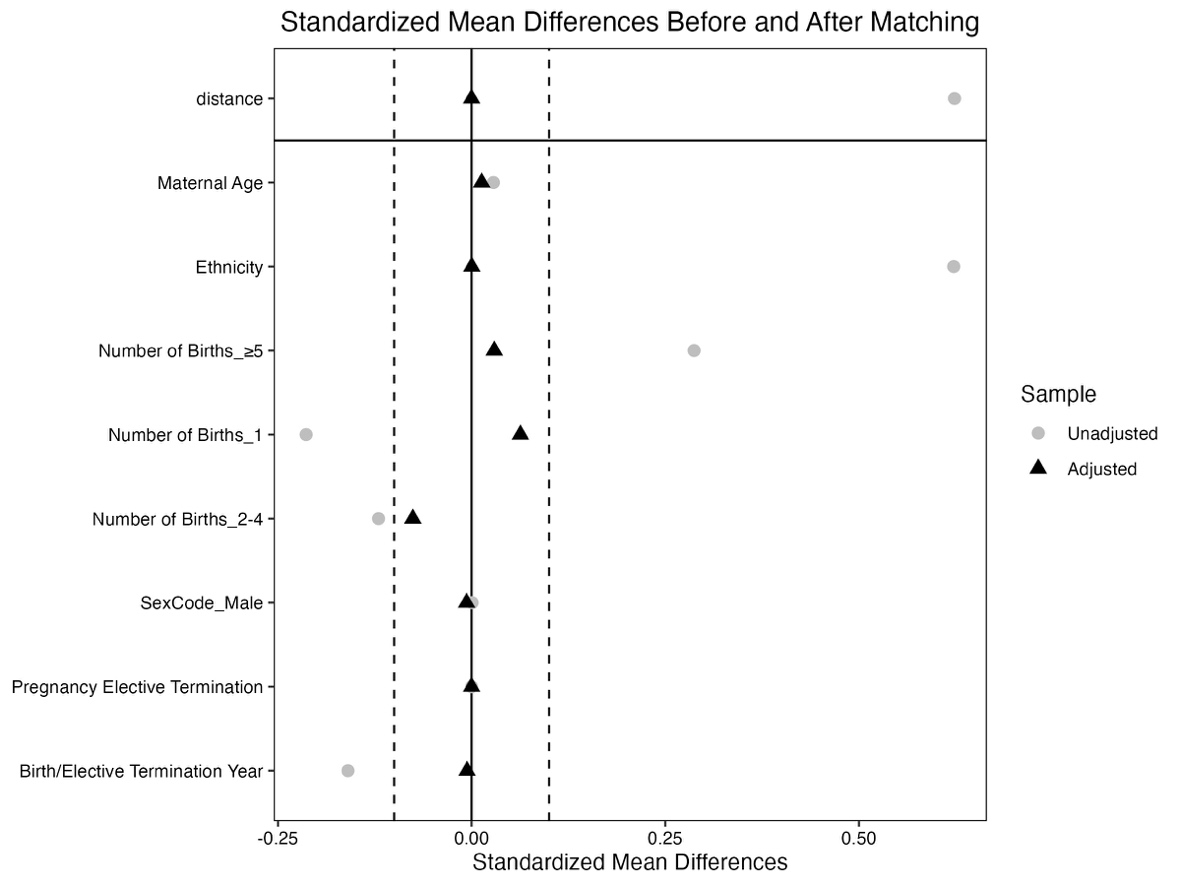


Figure S1. Standardized mean differences of baseline characteristics between exposed and unexposed groups before matching (unadjusted) and after matching (adjusted). Values closer to zero indicate better balance between groups, with the conventional threshold for adequate balance indicated by dashed lines at -0.1 and 0.1.

Table S5. Late adverse pregnancy outcomes following second-trimester exposure to doxycycline

| **Adverse 3rd trimester pregnancy outcome** | **Third trimester exposure to doxycycline**  N = 112^1^ | **Unexposed**  N = 265,574^1^ | **Difference**^2^ | **p-value**^4^ |
| --- | --- | --- | --- | --- |
| Low birth weight | 10 (8.9%) | 20,563 (7.8%) | -0.04 | 0.65 |
| Very low birth weight | 6 (5.4%) | 3,397 (1.3%) | -0.23 | 0.003 |
| Apgar score at 1 min <7 | 3 (2.7%) | 10,574 (4.1%) | 0.08 | 0.63 |
| Apgar score at 5 min <7 | 1 (0.9%) | 1,815 (0.7%) | -0.02 | 0.54 |
| Perinatal death | 4 (3.6%) | 4,674 (1.8%) | -0.11 | 0.13 |
| Preterm delivery | 11 (9.8%) | 22,492 (8.5%) | -0.05 | 0.61 |
| ^1^n (%)  ^2^Standardized Mean Difference  ^3^CI = Confidence Interval  ^4^Pearson's Chi-squared test; Fisher's exact test | | | | |

**Table S6. Late adverse pregnancy outcomes following third-trimester exposure to doxycycline**

| **Adverse 2nd trimester pregnancy outcome** | **Second trimester exposure to doxycycline**  N = 134^1^ | **Unexposed**  N = 265,552^1^ |  | **Difference**^2^ | **p-value**^3^ |
| --- | --- | --- | --- | --- | --- |
| Low birth weight | 11 (8.2%) | 20,562 (7.8%) |  | -0.01 | 0.86 |
| Very low birth weight | 1 (0.7%) | 3,402 (1.3%) |  | 0.05 | 0.99 |
| Apgar score at 1 min <7 | 8 (6.1%) | 10,569 (4.1%) |  | -0.09 | 0.24 |
| Apgar score at 5 min <7 | 1 (0.8%) | 1,815 (0.7%) |  | -0.01 | 0.60 |
| Perinatal death | 5 (3.7%) | 4,673 (1.8%) |  | -0.12 | 0.08 |
| Preterm delivery | 6 (4.5%) | 22,497 (8.5%) |  | 0.16 | 0.09 |
| ^1^n (%)  ^2^Standardized Mean Difference  ^3^Pearson's Chi-squared test; Fisher's exact test | | | | | |
